# Awareness, Acceptance, and Uptake of HIV Pre-Exposure Prophylaxis among Men Who Have Sex with Men (MSM) in Northwestern China: A Cross-Sectional Study

**DOI:** 10.1007/s10461-025-04884-8

**Published:** 2025-09-29

**Authors:** Rui Zhao, Yuxuan Li, Shiyi He, Wendi Zhang, Jiajun Sun, Zeping Fang, Leilei Zhang, Shu Su, Jason J. Ong, Lei Zhang

**Affiliations:** 1 Shenzhen Hospital of Southern Medical University, Shenzhen, Guangdong China; 2 China-Australia Joint Research Center for Infectious Diseases, School of Public Health, Xi’an Jiaotong University Health Science Center, Xi’an, Shaanxi China; 3 Melbourne Sexual Health Centre, Alfred Health, Melbourne, VIC Australia; 4 Central Clinical School, Faculty of Medicine, Nursing and Health Sciences, Monash University, Melbourne, Australia; 5 Department of Epidemiology and Biostatistics, The Second Affiliated Hospital of Chongqing Medical University, Chong Qing, China; 6 Faculty of Infectious and Tropical Diseases, London School of Hygiene and Tropical Medicine, London, United Kingdom; 7 Phase I clinical trial research ward, The Second Affiliated Hospital of Xi’an Jiaotong University, No. 157 Xi Wu Road, 710004 Xi’an, China

**Keywords:** HIV, Men who have sex with men, PrEP, Risk perception, Risk discrepancy

## Abstract

We assessed the awareness, acceptance (willingness to use), and uptake of pre-exposure prophylaxis (PrEP), along with the gap between PrEP eligibility and self-perceived HIV risk among men who have sex with men (MSM) in Shaanxi Province, China in 2022. Participants completed surveys on PrEP, and were screened for HIV, syphilis, *Chlamydia trachomatis*, and *Neisseria gonorrhoeae*. PrEP eligibility was determined using Chinese PrEP guidelines, and participants’ self-perceived HIV risk was documented. Multivariable logistic regression Analysed factors influencing PrEP-related outcomes And the discrepancy between perceived and actual risk. Among 1,085 participants (median age: 32 years), 54.4% resided in urban areas, 71.1% identified as homosexual, And 60.8% held a college degree or higher. Although 87.0% were PrEP-eligible, only 28.5% were identified as high risk. PrEP awareness was 82.0%, yet only 8.8% had used it, And 3.3% currently used it. Despite 72.9% expressing willingness, uptake was hindered by high costs. Rural residence and lower education correlated with reduced awareness and acceptance, while greater income, more male partners, and recent STI testing predicted higher acceptance. Prior PEP use was strongly associated with PrEP use. Targeted efforts to improve affordability, accessibility, and education are crucial for better prevention results.

**Supplementary Information:**

The online version contains supplementary material available at 10.1007/s10461-025-04884-8.

## Introduction

 For men who have sex with men (MSM) at high risk of HIV infection, HIV pre-exposure prophylaxis (PrEP) recommended as a highly effective biomedical prevention method against acquiring HIV [1–3]. PrEP has been approved And recommended in China since 2020 [4]. Despite the approval of PrEP in China since 2020, significant gaps remain in its implementation. While MSM in some regions show relatively high willingness to adopt PrEP (60–80%) [5, 6], awareness remains low, with only 25% of MSM in rural areas aware of it [7]. Access is limited, especially in rural areas, where only 20–30% of high-risk individuals have access, compared to over 80% in major cities like Beijing and Shanghai [8]. Furthermore, integration of PrEP into public health programs is insufficient, with only 10–15% of healthcare providers offering it as part of routine HIV prevention services [9].

Due to the relatively late recommendation of PrEP in China and the lack of financial subsidies and policy support from the government, there are currently mixed reports on the knowledge and acceptance and willingness to use PrEP in Chinese MSM and other key populations who may benefit from using PrEP [10, 11]. and the actual uptake rate remains very low [12]. In 2022, a cross-sectional study among MSM in Northwest China found that 81.1% of MSM had heard of PrEP, but only 47.6% were willing to use it [13]. Research During 2013–2018 among MSM in Shanghai And Hong Kong found that PrEP coverage was only 2.5% [14] And 4% [15] among eligible MSM. In addition, since the approval of PrEP in China in 2020, there have been few surveys on the awareness, acceptance, and uptake of PrEP by the same group of MSM.

Other studies have reported that self-perceived risk of HIV infection influences individuals’ attention, willingness to accept, and actual use of PrEP [16–18]. However, the alignment between objective PrEP eligibility (defined by Chinese PrEP guidelines as having recent condomless anal sex, multiple partners, or STI diagnosis) and subjective risk perception among Chinese MSM remains unclear. This clinically important discrepancy between objective eligibility and subjective perception may lead to both inadequate PrEP coverage among high-risk individuals and unnecessary demand among lower-risk groups. Therefore, understanding and assessing the discrepancy is essential to guide health education and enhance their ability to accurately recognize their risk of HIV and proactively choose appropriate HIV prevention services. In China, the rate of new HIV infections among MSM in the northwest is close to the national average for MSM [19]. In view of this, we conducted a cross-sectional study in the northwest to investigate the awareness, acceptance and uptake of PrEP, as well as discrepancy between the eligibility for PrEP and self-perceived risk of HIV infection among MSM in China.

## Methods

### Study Design and Participants

This study was conducted from January to December in 2022 in Shaanxi Province, China. The recruitment and implementation of the study relied on the Tongjian Mutual Aid Workgroup, an MSM community with extensive experience in HIV/AIDS prevention and education. Participants were invited to participate in on-site surveys and were screened for HIV, syphilis, chlamydia and gonorrhoea at the outreach community centre. The inclusion criteria for participants were those: aged 18 years or older; assigned male at birth And engaged in male-male sexual behaviors in the last 3 months; self-reported negative or unknown HIV status; signed An informed consent form And expressed willingness to participate in the study. Exclusion criteria included severe mental illness, language barriers, intellectual disabilities, or individuals deemed ineligible for the study by the investigators. Our study provided a subsidy of CNY 50 for each participant included in the study.

### Survey and HIV/STI Testing

MSM who participated in our study were required to be reviewed by staff for compliance with inclusion criteria. Then the included participants completed an online self-administered survey by scanning the QR code on the electronic recruitment poster. The questions in the survey included: demographic characteristics; PrEP-related questions: awareness (“Have you ever heard of PrEP for HIV prevention?”), acceptance (“Are you willing to use PrEP for HIV prevention?”) and uptake (“Are you using or have you ever used PrEP for HIV prevention?”) [20, 21]; self-reported HIV/STI testing, STI diagnosis, and sexual behaviors; alcohol consumption and recreational drug use. To assess reasons for rejecting PrEP, participants were asked to select from a set of predefined response options. In addition, open-ended responses were also allowed for participants to elaborate further if they wished. The anonymous online survey only allowed one unique IP address to fill out the survey to avoid multiple entries by the same participant. Table S1 provides a detailed list of the questions included in the survey. The survey questions were based on previously validated instruments [22, 23].

After completing the survey, trained staff performed STI testing for the participants, including HIV, syphilis, chlamydia and gonorrhoea. We collected fingerpick blood of the participants, and conducted rapid tests for HIV (HIV Antibody (HIV 1/2) test kit produced by Wanfu Biological Co) and syphilis (syphilis spirochete antibody test kit produced by Intect Products Inc Co). For participants with positive results of screening, confirmatory laboratory of HIV and syphilis testing were conducted by the designated Center for Disease Control and Prevention (CDC). We collected swab specimens from three sites (oropharynx, anus, and urethra), and used nucleic acid amplification assay (gonococcal and chlamydia trachomatis nucleic acid detection kits produced by Shanghai Lianzu Biotechnology Co.). Shaanxi inspection company was responsible for the transfer, storage and testing of chlamydia and gonorrhoea.

### Assessment of the Eligibility for PrEP and Self-Perceived Risk of HIV Infection

Part A: We applied the criteria in the PrEP guideline in China to evaluate their eligibility for PrEP use among Chinese MSM [4]. The criteria contained five questions: ⅰ. Have you had sex with a male or female partner without a condom in the past 3 months? ⅱ. Have you injected illegal drugs And shared needles in the past 3 months? ⅲ. Have you had a sexual partner who was infected with HIV? ⅳ. Have you been newly diagnosed with STI, such as syphilis, gonorrhoea, or chlamydia? ⅴ. Have you used or are you willing to use PrEP or HIV post-exposure prophylaxis (PEP) to prevent HIV infection through sexual or intravenous injection routes? Among the five questions, sexual behavior could include anal and vaginal sex, and if any one of the questions was answered “yes”, it would be considered “high-risk behaviors for HIV exposure”. To avoid recall bias, the study further shortened the evaluation period from the past 6 months in the expert consensus to the past 3 months. Moreover, considering that the participants may have STIs without showing symptoms, the diagnosis of the fourth question was based on the results of the on-site sampling test for syphilis, chlamydia and gonorrhoea.

Part B: To evaluate the self-perceived risk of HIV infection, a risk assessment scale from Brazil was adopted [24], and the participants were asked “How likely do you think you are to be infected with HIV?” And were required to select a number from 0 to 10 based on their self-perceived risk of HIV infection, with 0 representing “not at all likely” And 10 representing “completely likely”. Based on the participants’ scores, those who scored ≥ 5 was classified as having a high self-perceived risk, while those who scored < 5 was classified as having a low self-perceived risk. This dichotomous classification was adapted from a prior study conducted in Thailand, which simplified the original scale to facilitate analysis [25].

Then by combing the results assessed above, the discrepancy between the eligibility for PrEP and self-perceived risk of HIV infection of the participants was defined as an individual who has high HIV risk (determined but Part A) with low self-perception (determined by Part B), or vice vera.

### Statistical Analysis

After cleaning the data, SPSS 26.0 statistical software was used for statistical analysis. The PrEP awareness and uptake rates, and the discrepancy rate between the eligibility for PrEP and self-perceived risk of HIV infection were calculated for all included participants, and the acceptance rate was calculated for the participants who had never used PrEP. The chi-square test or Fisher’s exact method was used to analyze the relationship between various factors and the awareness, acceptance and uptake of PrEP, as well as the discrepancy rate between the eligibility for PrEP and self-perceived risk of HIV infection among participants. Factors with *P* < 0.1 in the above univariate analysis were selected for further multivariable logistic regression analysis. We used the Forced Entry method, with *P* < 0.05 as the criterion for selection of variables and *P* > 0.1 as the criterion for exclusion of variables, and *P* < 0.05 was considered as statistically different.

## Results

### Overview of Demographics

Our recruitment process initially identified 1200 potential participants through social media advertisements And community-based organizations. After preliminary screening, 1212 MSM met the basic inclusion criteria And were enrolled in the study. Of these, 127 were excluded according to the exclusion criteria And the remaining 1085 were included in the analysis (response rate: 89.5%), with a median age of 32 years (IQR: 27–39 years). Of the participants included, 54.4% (599/1085) resided in urban areas, 71.1% (771/1085) identified as homosexual, 51.0% (553/1085) had disclosed their sexual orientation to others, 30.2% (327/1085) were married with females, 60.8% (659/1085) had the degree of university/college And above, 30.0% (325/1085) were freelancers, And 18.1% (196/1085) with monthly income < CNY 3000.

Of the included participants, 4.6% (50/1085) were tested positive for syphilis, 12.6% (137/1085) for chlamydia (oropharyngeal: 0.8% (9/1085), urethral: 1.6% (17/1085), Anal: 11.0% (119/1085)), And 13.2% (143/1085) for gonorrhoea (oropharyngeal: 7.8% (85/1085), urethral: 1.3% (14/1085), Anal: 4.8% (52/1085)), respectively. Further, 76.5% (830/1085) And 74.3% (806/1085) had tested for HIV or other STIs in the last 3 months, respectively; 11.9% (129/1085) had ever been diagnosed with STIs, 7.1% (77/1085) reported Any current symptoms of STIs, And 13.5% (147/1085) had used PEP for HIV prevention. When asked about their behaviors in the last 3 months, 4.4% (129/1085) had sexual partners with HIV, 4.3% (47/1085) And 12.2% (132/1085) had sex with foreigners or women, respectively; 20.4% (221/1085) had more than 5 sexual partners, with a median number of 2 (IQR: 1–5), And 46.3% (502/1085) had casual or commercial partners in the last sexual act; 5.8% (63/1085) drank alcohol > 3 times per week, And 11.3% (123/1085) had used recreational drugs (e.g., methamphetamine, Rush, Ketamine) during last male-to-male sex (Table 1).

**Table 1:** Demographic characteristics of study participants (*N* = 1085)

Characteristics	*n*	%
*Demographic*		
Age (years)		
Median (IQR)	32 (27–39)	…
Age group (years)		
18–25	177	16.3
26–35	535	49.3
36–45	254	23.4
> 45	119	11.0
Residence		
Urban	590	54.4
Town	271	25.0
Rural	224	20.6
Sexual orientation		
Homosexual	771	71.1
Bisexual or other	314	28.9
Disclosure of sexual orientation to others		
Yes	553	51.0
No	532	49.0
Marital status (with female)		
Single	673	62.0
Married	327	30.2
Divorced/Widowed	85	7.8
Education		
Middle school and below	149	13.7
High school/Junior college	277	25.5
University/College and above	659	60.8
Occupation		
Freelancer	325	30.0
Corporate employees	179	16.5
Individual entrepreneurship	305	28.1
Government official	188	17.3
Unemployed/Retired/Student	88	8.1
Income (CNY/month)		
< 3000	196	18.1
3000–6000	481	44.3
> 6000	408	37.6
*HIV/STI testing*,* infection and behaviours*		
HIV testing in the last 3 months		
Yes	830	76.5
No	255	23.5
Other STI testing in the last 3 months		
Yes	806	74.3
No	279	25.7
Diagnosed with STIs before		
Yes	129	11.9
No	778	71.7
Not sure/Refuse to answer	178	16.4
Any current STI symptoms		
Yes	77	7.1
No	998	92.0
Not sure/Refuse to answer	10	0.9
Ever used PEP (post-exposure prophylaxis) for HIV prevention		
Yes	147	13.5
No	938	86.5
Any sexual partners with HIV infection in the last 3 months		
Yes	48	4.4
No	772	71.2
Not sure/Refuse to answer	265	24.4
Have sex with foreigners in the last 3 months		
Yes	47	4.3
No	997	91.9
Not sure/Refuse to answer	41	3.8
Have sex with women in the last 3 months		
Yes	132	12.2
No	919	84.7
Not sure/Refuse to answer	34	3.1
Number of male sexual partners in the past 3 months		
Median (IQR)	2 (1–5)	…
< 3	662	61.0
3–5	202	18.6
> 5	221	20.4
Male sexual partner in the last sexual act		
Regular sex partner (boyfriend)	374	34.5
Long-term fuck buddy	209	19.2
Casual or commercial sex partner	502	46.3
Usual frequency of alcohol drinking (per week)		
Never	544	50.1
Sometimes (< 3 times)	478	44.1
Always (≥ 3 times)	63	5.8
Ever used prohibited drugs during last male-to-male sex		
Yes	123	11.3
No	962	88.7
Self-perceived risk of HIV infection		
High	269	24.8
Low	816	75.2
*PrEP related*		
Have heard of PrEP		
Yes	890	82.0
No	195	18.0
Have heard of daily PrEP		
Yes	846	78.0
No	239	22.0
Have heard of on-demand PrEP		
Yes	714	65.8
No	371	34.2
Have heard of long-acting injectable PrEP		
Yes	424	39.1
No	661	60.9
Stage of PrEP use		
Currently using	36	3.3
Ever used	95	8.8
Never used and willing to use	681	62.8
Never used and not willing to use	253	23.3
Refuse to answer	20	1.8
Willingness to pay for PrEP (CNY/month)		
Median (IQR)	300 (200–800)	…
< 500	725	66.8
500–1000	265	24.4
1000–2000	73	6.7
> 2000	22	2.0

*CNY* Chinese Yuan, *IQR* interquartile range, *PrEP* pre-exposure prophylaxis, *STI* sexually transmitted infections

### Awareness, Acceptance and Uptake of PrEP among MSM

Of the participants included, 82.0% (890/1085) had heard of PrEP, 8.8% (95/1085) had used but were currently off PrEP, And 3.3% (36/1085) were currently using PrEP. Further, 72.9% (681/934) had never used but were willing to use PrEP. If available, the median willingness-to-pay for PrEP per month was CNY 300 (IQR: CNY 200–800), with 66.8% (725/1085) willing to pay < CNY 500 (Table 1). Of the 253 participants who had never used and were unwilling to use PrEP, “Drugs are too expensive”, “No need for medication” and “Don’t know where to buy PrEP” were the most cited reasons for their reluctance to use PrEP (Fig. S1). For the 95 participants who had used but were currently off PrEP, “No need for medication”, “Don’t know where to buy PrEP”, “Concerned about the side effects of PrEP” were the most cited reasons for discontinuing PrEP use (Fig. S2).

Multivariable logistic regression indicated that the following factors were significantly associated with the awareness of PrEP among MSM: living in rural areas (vs. urban areas, OR: 0.33, 95% CI: 0.20–0.56, *P* < 0.001), had the degree of university/college and above (vs. middle school And below, OR: 1.95, 95% CI: 1.09–3.52, *P* = 0.025), had not disclosed sexual orientation to others (vs. disclosed sexual orientation, OR: 0.46, 95% CI: 0.29–0.74, *P* = 0.001), had not been diagnosed with STIs (vs. had been diagnosed with STIs, OR: 0.38, 95% CI: 0.18–0.84, *P* = 0.017), had sex with casual or commercial partner in the last sexual act (vs. with boyfriend, OR: 0.53, 95% CI: 0.34–0.84; *P* = 0.006) (Table 2, see the results of univariate analysis in Table S2).

**Table 2:** Factors associated with awareness, acceptance, and uptake of PrEP, and discrepancy between the eligibility for PrEP and self-perceived risk of HIV among men who have sex with men in China

	Awareness (*n* = 1085)		Acceptance (*n* = 934)		Uptake (*n* = 1085)		Discrepancy (*n* = 1085)	
	OR (95% CI)	P*	OR (95% CI)	P*	OR (95% CI)	P*	OR (95% CI)	P*
Residence								
Urban	Ref.		Ref.				Ref.	
Town	1.06 (0.64–1.77)	0.816	0.72 (0.44–1.18)	0.190			1.66 (1.12–2.46)	0.011
Rural	0.33 (0.20–0.56)	< 0.001	0.49 (0.28–0.85)	0.010			0.97 (0.63–1.48)	0.872
Sexual orientation								
Homosexual							Ref.	
Bisexual and other							1.57 (1.12–2.20)	0.009
Disclosure of sexual orientation to others								
Yes	Ref.							
No	0.46 (0.29–0.74)	0.001						
Marital status (with female)								
Single					Ref.			
Married					0.87 (0.43–1.76)	0.690		
Divorced/Widowed					0.22 (0.06–0.79)	0.020		
Education								
Middle school and below	Ref.		Ref.				Ref.	
High school/Junior college	1.22 (0.70–2.13)	0.495	2.24 (1.20–4.17)	0.011			2.24 (1.37–3.66)	0.001
University/College and above	1.95 (1.09–3.52)	0.025	2.32 (1.24–4.33)	0.008			2.13 (1.30–3.48)	0.003
Occupation								
Freelancer	Ref.		Ref.				Ref.	
Corporate Employees	1.17 (0.66–2.07)	0.592	1.59 (0.89–2.86)	0.119			0.92 (0.59–1.42)	0.696
Individual Entrepreneurship	2.72 (1.57–4.68)	< 0.001	2.95 (1.71–5.10)	< 0.001			1.77 (1.18–2.65)	0.006
Government official	1.24 (0.71–2.17)	0.449	1.45 (0.81–2.60)	0.217			0.78 (0.51–1.20)	0.254
Unemployed/Retired/Student	1.93 (0.82–4.52)	0.131	4.71 (1.86–11.94)	0.001			2.34 (1.17–4.67)	0.016
Income (CNY/month)								
< 3000			Ref.					
3000–6000			2.36 (1.34–4.17)	0.003				
> 6000			2.39 (1.20–4.75)	0.013				
Other STI testing in the last 3 months								
Yes			Ref.				Ref.	
No			0.56 (0.35–0.89)	0.015			0.65 (0.43–0.99)	0.049
Diagnosed with STIs before								
Yes	Ref.							
No	0.38 (0.18–0.84)	0.017						
Not sure/Refuse to answer	0.74 (0.30–1.83)	0.512						
Ever used PEP against HIV infection								
Yes					Ref.			
No					0.01 (0.01–0.02)	< 0.001		
Any sexual partners with HIV infection in the last 3 months								
Yes	Ref.							
No	0.25 (0.05–1.21)	0.085						
Not sure/Refuse to answer	0.10 (0.02–0.49)	0.005						
Number of male sexual partners in the past 3 months								
< 3			Ref.				Ref.	
3–5			0.84 (0.48–1.46)	0.534			1.13 (0.76–1.68)	0.545
> 5			2.55 (1.44–4.50)	0.001			2.04 (1.35–3.09)	< 0.001
Male sexual partner in the last sexual act								
Regular sex partner (boyfriend)	Ref.		Ref.				Ref.	
Long-term fuck buddy	1.38 (0.70–2.73)	0.355	1.89 (0.83–4.30)	0.129			1.13 (0.71–1.80)	0.595
Casual or commercial sex partner	0.53 (0.34–0.84)	0.006	0.18 (0.12–0.29)	< 0.001			0.51 (0.36–0.72)	< 0.001
Usual frequency of alcohol drinking (per week)								
Never	Ref.		Ref.		Ref.			
Sometimes (< 3 times)	2.50 (1.55–4.02)	< 0.001	2.24 (1.37–3.68)	0.001	0.98 (0.50–1.92)	0.961		
Always (≥ 3 times)	2.83 (1.06–7.53)	0.038	1.78 (0.76–4.20)	0.187	0.16 (0.03–0.83)	0.028		
Ever used prohibited drugs during last male-to-male sex								
Yes							Ref.	
No							4.16 (2.49–6.95)	< 0.001

*CNY* Chinese Yuan, *OR* odds ratio, *PEP* post-exposure prophylaxis, *PrEP* pre-exposure prophylaxis, *STI* sexually transmitted infections

^*^Multivariable logistic regression

The following factors were significantly associated with the acceptance of PrEP among MSM: living in rural areas (vs. urban areas, OR: 0.49, 95% CI: 0.25–0.85, *P* = 0.01), had the degree of university/college and above (vs. middle school And below, OR: 2.32, 95% CI: 1.24–4.33, *P* = 0.008), monthly income > CNY 6000 (vs. < CNY 3000, OR: 2.39, 95% CI: 1.20–4.75, *P* = 0.013), had not tested STIs in the last 3 months (vs. tested STIs, OR: 0.56, 95% CI: 0.35–0.89; *P* = 0.015), had > 5 male sexual partners in the last 3 months (vs. <3 partners, OR: 2.55, 95% CI: 1.44–4.50; *P* = 0.001), had sex with casual or commercial partner in the last sexual act (vs. with boyfriend, OR: 0.18, 95% CI: 0.12–0.29; *P* < 0.001) (Table 2, see the results of univariate analysis in Table S2).

The following factors were significantly associated with the uptake of PrEP among MSM: divorced/widowed (vs. single, OR: 0.22, 95% CI: 0.06–0.79; *P* = 0.02), had not used PEP (vs. used PEP, OR: 0.01, 95% CI: 0.01–0.02; *P* < 0.001), and drank alcohol > 3 times per week (vs. never drank, OR: 0.16, 95% CI: 0.03–0.83; *P* = 0.028) (Table 2, see the results of univariate analysis in Table S2).

### Discrepancy Between the Eligibility for PrEP and Self-Perceived Risk of HIV Infection among MSM

**Fig. 1:**
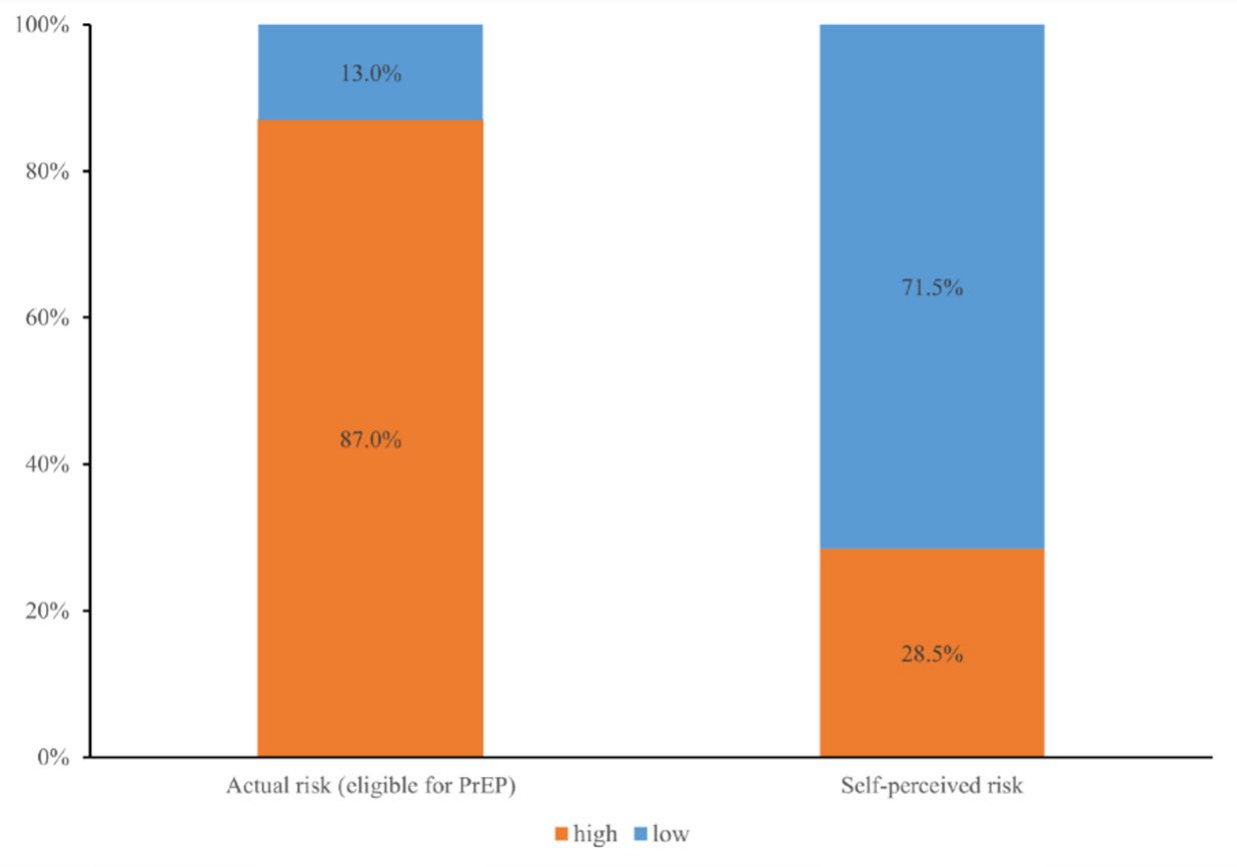
Discrepancy between the eligibility for PrEP and self-perceived risk of HIV infection among MSM

Of the participants included, according to the suitability criteria of PrEP guideline in China, 87.0% (944/1085) were assessed as being at high risk for HIV infection And eligible for PrEP use; however, only 28.5% (269/944) self-perceived as being at high risk for HIV infection. This revealed a considerable discrepancy between the eligibility for PrEP and self-perceived risk of HIV infection among MSM (Fig. 1).

Multivariate logistic regression indicated that the following factors were significantly associated with the discrepancy among MSM: living in towns (vs. urban areas, OR: 1.66, 95% CI: 1.12–2.46, *P* = 0.011), bisexual (vs. homosexual, OR: 1.57, 95% CI: 1.12–2.20, *P* = 0.009), had the degree of university/college and above (vs. middle school And below, OR: 2.13, 95% CI: 1.30–3.48, *P* = 0.003), had not tested STIs in the last 3 months (vs. tested STIs, OR: 0.65, 95% CI: 0.43–0.99, *P* = 0.049), had > 5 male sexual partners in the last 3 months (vs. <3 partners, OR: 2.04, 95% CI: 1.35–3.09, *P* < 0.001), had sex with casual or commercial partner in the last sexual act (vs. with boyfriend, OR: 0.51, 95% CI: 0.36–0.72, *P* < 0.001), and had not used prohibited drugs in the last male-to-male sex (vs. used prohibited drugs, OR: 4.16, 95% CI: 2.49–6.95, *P* < 0.001) (Table 2, see the results of univariate analysis in Table S3).

## Discussion

Two years after the approval of PrEP in China, we investigated the current awareness, acceptance and uptake of PrEP among Chinese MSM. Our analysis found that a high proportion of MSM was already aware of (82.0%) and willing to use PrEP (72.9%); however, the uptake of PrEP remained low, with only 8.8% having ever used but currently off PrEP, And 3.3% currently using PrEP, respectively. Further, our study also evaluated the discrepancy between the eligibility for PrEP And self-perceived risk of HIV infection among Chinese MSM, And found 87.0% of the included participants were assessed as being at high risk And eligible for PrEP use; however, only 28.5% perceived themselves as being at high risk for HIV infection.

Similar to our results, a survey conducted in Shaanxi province found that 81.1% of MSM had heard of PrEP [13]; a 2022 meta-analysis also showed that the average acceptance of PrEP was 68.4% in China, while MSM had the highest acceptance (72.4%) [10]. Our Analysis indicated that PrEP awareness And acceptance were lower among MSM who lived in rural areas and had low levels of education. In 2022, a study in Shandong province also found that PrEP awareness was lower among rural MSM, suggesting that MSM in rural areas were less aware of PrEP than those in urban areas due to a lack of healthcare resources and access to HIV-related information and services, and therefore had a lower willingness to accept it [26].

In addition to urban-rural differences, our analysis also identified key factors influencing PrEP awareness and acceptance among MSM. We found that MSM with increasing age, higher education levels, and peer recognition had better awareness of PrEP. These factors have been shown in previous studies to significantly impact awareness, as MSM with more education and social support are more likely to be informed about PrEP [27]. Price was Another critical factor affecting the acceptance of PrEP. Our results demonstrated that 67% of MSM were willing to pay < CNY 500 per month for PrEP, and “Drugs are too expensive” was the first reason for MSM reluctant to use PrEP. Wang et al. also reported that MSM were willing to use PrEP when self-funded was only 7.7%, but their willingness to accept would rise to 45.2% if it were provided free of charge [28]. Consistent with the results of studies in Beijing and Shanghai During 2018–2022 [29, 30], we also found that PrEP awareness was higher among MSM diagnosed with STIs, and acceptance was higher among those with more male sexual partners and recent STI tests. All of the above results indicated that MSM are aware that high-risk behaviours result in an elevated risk of HIV infection, and would protect themselves by actively accessing PrEP-related information and services. Therefore, Chinese health service providers should formulate appropriate publicity strategies for MSM in different regions and characteristics with high-risk behaviours, while also reducing PrEP costs to increase both awareness and uptake across China.

Despite the high awareness And acceptance of PrEP among Chinese MSM, the actual uptake of PrEP remains low, suggesting a significant gap in PrEP usage. Similar to our findings, a cross-sectional survey conducted in Hefei, Chengdu, And Guangzhou in 2018 also found that although 67.2% of MSM had An actual need for PrEP, only 4.3% of MSM had ever used PrEP [12]. By the end of 2023, PrEP has been approved for use in 70 countries worldwide [31]. Compared to other countries, the promotion of PrEP in China has remained slow, with only a few PrEP demonstration projects currently underway [32, 33]. The expansion of PrEP in China could be accelerated by assimilating And capitalizing on the successful experiences of other countries. As Another middle-income country in Asia and Pacific region, Thailand launched a demonstration PrEP project in 2016 called ‘Princess PrEP’, a key population-led PrEP program implemented in six priority provinces in the country [34]. Through training and certifying lay providers who are also members of the communities they serve, high-quality and non-judgmental PrEP and other HIV services have been provided along the HIV cascade in community-based organizations without stigma and discrimination [35]. As a result, Thailand has achieved 31% of PrEP coverage among eligible MSM by 2022 [36]. Other studies suggested that PrEP utilization among key populations can also be successfully promoted by services such as facilitating the efficiency of PrEP services through a model that combines online counselling and offline services [37, 38]. A demonstration project that tested 76.5% of MSM for HIV after providing online counselling And risk assessment in conjunction with An offline setting at a healthcare facility, and ultimately prompted up to 53.2% of participants to begin using PrEP [39]. China can further increase the accessibility and utilization of PrEP among MSM by strengthening the construction of PrEP-related web-based counselling services, as well as increasing PrEP services in offline community hospitals, and increasing PrEP-related publicity and education by setting up mobile clinics or testing sites [40].

Similar to the results of our analysis, discrepancy between the eligibility for PrEP and self-perceived risk of HIV are also common in other studies. The iPrEX study conducted in US During 2011–2012 evaluated the suitability of PrEP for MSM And their self-perception of HIV infection, demonstrating that while 80.3% met the criteria for PrEP use, 78% perceived low risk of HIV infection and no need for PrEP use [41]. A PrEP demonstration project in Thailand in 2015–2016 found that over 80% of those who perceived themselves to be at low risk of HIV infection were actually eligible for PrEP (i.e., at high risk of HIV infection) [25]. Those results suggested that a high proportion of high-risk MSM underestimate their risk of HIV infection both in high- and middle-/low-income countries. Our study uncovered that those with higher levels of education were more likely to have misperception of their HIV risk than those with lower education levels, suggesting that even with higher levels of literacy, MSM may not be able to objectively And accurately quantify their actual risk of acquiring HIV. This misperception was also found among MSM with more male sexual partners And those who had tested for STIs in the last 3 months when it comes to PrEP awareness And acceptance in our study. However, Another study in Thailand also found that nearly 50% of those who reported not using condoms throughout sex, having multiple sex partners, having group sex, or having been diagnosed with STIs in the past perceived themselves to be at no or only a low risk of HIV infection [42]. Therefore, it is essential for health service workers to guide health education for MSM and enhance their ability to accurately evaluate their actual risk of HIV infection and proactively seek appropriate preventive measures such as PrEP and HIV/STI testing.

Our study has several limitations. First, the study was conducted in Xi’an, and the findings cannot be generalized to the entire Chinese MSM population due to significant regional variations in the socio-demographic characteristics of this population. Second, our recruitment from the Tongjian Mutual Aid Workgroup may have led to an over-sampling of health-literate MSM, as evidenced by the high baseline PrEP awareness (82.0%) and education levels (60.8% with college education). This may introduce bias, as the sample may not accurately represent the broader MSM population, particularly those with limited healthcare access or lower levels of education. While the sample reflects China’s current focus on PrEP implementation among engaged populations, these results may not be fully generalizable to MSM who are less engaged or have less access to HIV-related information and services. Third, our study only investigated awareness, acceptance and uptake of PrEP, while the evaluation of adherence and retention rates after PrEP use were not included. This omission was motivated by the fact that China is currently in the early stage of PrEP implementation, and the coverage among MSM remains low. Additionally, there are no established guidelines available to facilitate follow-up testing of PrEP users to ensure their adherence and retention rates. Therefore, policy makers need to develop and refine norms for PrEP use and conduct further research on adherence and retention to gain a more comprehensive understanding of PrEP implementation in China. Furthermore, as we conducted a cross-sectional study, causal relationships could not be drawn for the associated factors affecting PrEP awareness, acceptance and uptake among MSM, suggesting the need for further longitudinal studies.

## Conclusions

Chinese MSM had relatively high levels of PrEP awareness and acceptance, yet the uptake remained low. We uncovered a large discrepancy between their eligibility for PrEP and self-perceived risk of HIV infection. Therefore, Chinese health service providers should formulate appropriate publicity strategies to increase accurate awareness of PrEP among MSM, as well as increase the affordability of PrEP by appropriately lowering the price of the drug to increase their acceptance of PrEP. Tools for MSM to more accurately evaluate their actual risk of HIV infection and offer HIV prevention if needed is urgently needed.

## Supplementary Information

Below is the link to the electronic supplementary material.

Supplementary Material 1

## Data Availability

The data that support the fndings of this study are available from the corresponding author upon reasonable request.
