# Supplementary material for: Awareness, Acceptance, and Uptake of HIV Pre-Exposure Prophylaxis among Men Who Have Sex with Men (MSM) in Northwestern China: A Cross-Sectional Study
Source: AIDS Behav. 2025 Sep 29;30(2):429–39. doi: 10.1007/s10461-025-04884-8 (PMC12929275; doi:10.1007/s10461-025-04884-8)
Supplement: Supplementary file 1 — Supplementary Material 1 [file 10461_2025_4884_MOESM1_ESM.docx]

**Supplementary materials**

**
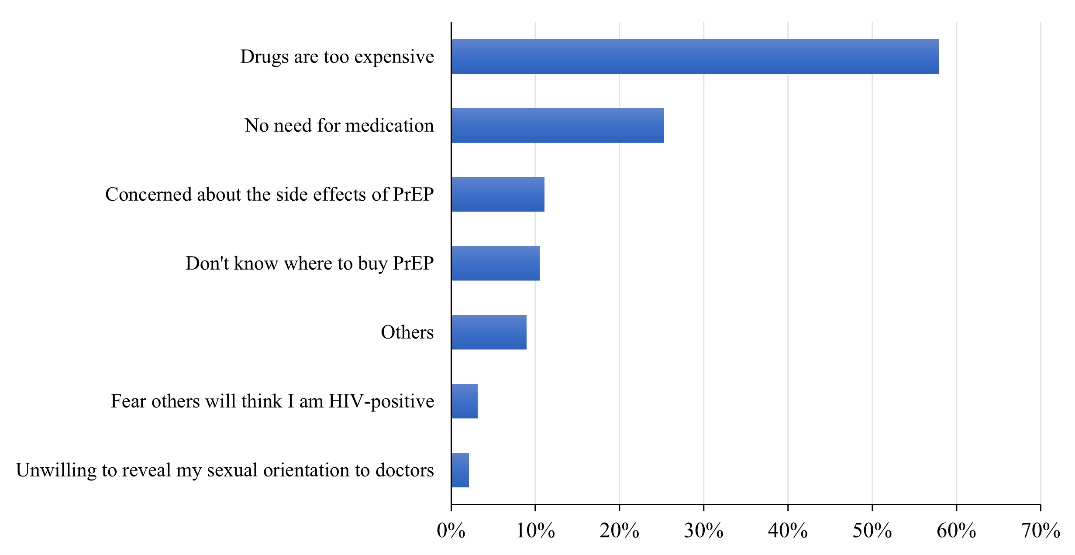
**

**Figure S1.** Reasons for not willing to use PrEP among MSM who have never used PrEP


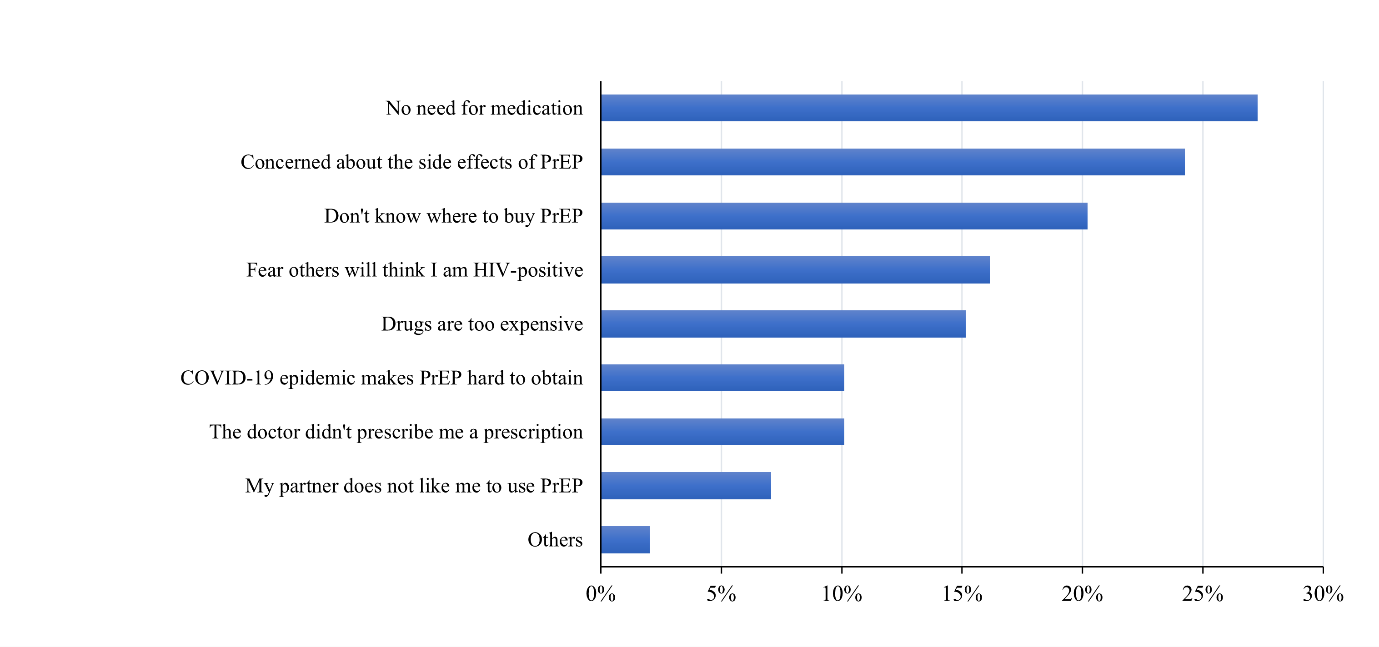


**Figure S2.** Reasons for discontinuing PrEP among who have ever used PrEP

**Table S1.** Survey instrument

| **Question** | **Options** |
| --- | --- |
| **Section 1: Demographic Characteristics** |  |
| **1. Date of Birth** | Text input (YYYY-MM) |
| **2. Current Residence** | □ Rural |
|  | □ Town |
|  | □ Urban |
| **3. Sexual Orientation** | □ Homosexual |
|  | □ Bisexual |
|  | □ Other (Specify: ______) |
| **4. Ethnicity** | □ Han Chinese |
|  | □ Other (Specify: ______) |
| **5. Marital Status (with female)** | □ Single |
|  | □ Married |
|  | □ Divorced |
|  | □ Widowed |
| **6. Education Level** | □ Junior high or below |
|  | □ High school/vocational |
|  | □ College |
|  | □ Postgraduate or above |
| **7. Occupation** | □ Student |
|  | □ Corporate employee |
|  | □ Government employee |
|  | □ Self-employed |
|  | □ Freelancer |
|  | □ Retired |
|  | □ Unemployed |
|  | □ Other (Specify: ______) |
| **8. Monthly Income (CNY)** | □ <3,000 |
|  | □ 3,001-6,000 |
|  | □ 6,001-9,000 |
|  | □ >9,000 |
| **9. Have you disclosed your sexual orientation ("come out") to: (Multiple choice)** | □ Family |
|  | □ Friends |
|  | □ Supervisors/colleagues |
|  | □ Doctors/nurses |
|  | □ CDC/community workers |
|  | □ Other (______) |
|  | □ Not disclosed to anyone |
|  | □ Hepatitis B |
|  | □ Hepatitis C |
|  | □ Other (Specify: ______) |
|  | □ Unsure |
|  | □ Refuse to answer |
| **Section 2: PrEP-related questions** |  |
| **10. Have you ever heard of PrEP for HIV prevention?** | □ Yes |
|  | □ No |
| **11. Are you willing to use PrEP for HIV prevention?** | □ Yes |
|  | □ No |
|  | □ Unsure |
| **12. What are your reasons for unwillingness to use PrEP? (Select all that apply)** | □ Don't know where to obtain PrEP |
|  | □ COVID-19 made PrEP inaccessible |
|  | □ No perceived need for medication |
|  | □ Concerned about side effects |
|  | □ Doctor won't prescribe PrEP |
|  | □ Unwilling to disclose sexual orientation to prescribing doctor |
|  | □ Medication cost is too high |
|  | □ Fear of stigma (others assuming HIV+ status or promiscuity) |
|  | □ Other (please specify: _______) |
| **13. Are you using or have you ever used PrEP for HIV prevention?** | □ Currently using |
|  | □ Previously used |
|  | □ Never used |
| **Section 3: Self-reported HIV/STI testing, STI diagnosis, and sexual behaviors** | |
| **14. Have you ever been diagnosed with an STI?** | □ Yes |
|  | □ No (Skip to Q15) |
|  | □ Unsure (Skip to Q15) |
|  | □ Refuse to answer (Skip to Q15) |
| **15. Which STIs have you had? (Multiple choice)** | □ HIV |
|  | □ Syphilis |
|  | □ Gonorrhea |
|  | □ Genital warts |
|  | □ Nonspecific urethritis |
|  | □ Hepatitis B |
|  | □ Hepatitis C |
|  | □ Other (Specify: ______) |
|  | □ Unsure |
|  | □ Refuse to answer |
| **16. Do you currently have STI symptoms?** | □ Yes |
|  | □ No |
|  | □ Refuse to answer |
| **17. Have any partners (male/female) been HIV positive?** | □ Yes |
|  | □ No |
|  | □ Unsure |
| **18. Had sex with foreigners? (including during international travel)** | □ Yes |
|  | □ No |
|  | □ Unsure |
|  | □ Refuse to answer |
| **19. Had sex with women? (including kissing, oral, anal or vaginal sex)** | □ Yes |
|  | □ No |
|  | □ Unsure |
|  | □ Refuse to answer |
| **20. Condom use with women? (for vaginal/anal sex)** | □ Always use |
|  | □ Sometimes use |
|  | □ Never use |
|  | □ No vaginal/anal sex occurred |
|  | □ Unsure |
|  | □ Refuse to answer |
| **21. Condom use as insertive partner with men? (top position)** | □ Always use |
|  | □ Sometimes use |
|  | □ Never use |
|  | □ Didn't occur |
|  | □ Unsure |
|  | □ Refuse to answer |
| **22. Condom use as receptive partner with men? (bottom position)** | □ Always use |
|  | □ Sometimes use |
|  | □ Never use |
|  | □ Didn't occur |
|  | □ Unsure |
|  | □ Refuse to answer |
| **Section 4: alcohol consumption and recreational drug use** | |
| **23. How often do you typically drink alcohol?** | □ Never drink |
|  | □ Occasionally (<1 time/week) |
|  | □ 1-2 times/week |
|  | □ 3-4 times/week |
|  | □ 5-6 times/week |
|  | □ Daily |
| **24. In past 3 months, how often did you consume >5 drinks in one session? (5 drinks ≈ 1.7L beer/710mL wine/213mL liquor)** | □ 1-2 times total |
|  | □ ~1 time/month |
|  | □ ~1 time/week |
|  | □ >1 time/week |
|  | □ Daily |
|  | □ Never |
| **25. Have you ever used illicit drugs? (including recreational use)** | □ Yes |
|  | □ No |
| **26. Have you ever injected illicit drugs?** | □ Yes |
|  | □ No |
|  | □ Unsure |
| **27. When did you last inject drugs?** | □ Never injected |
|  | □ Within 3 months |
|  | □ 3-12 months ago |
|  | □ >12 months ago |
|  | □ Unsure |
|  | □ Refuse to answer |
| **28. In past 3 months, did you share needles when injecting?** | □ Yes |
|  | □ No |
|  | □ Refuse to answer |

**Table S2.** Univariate analysis of factors associated with awareness, acceptance, and uptake of PrEP, and discrepancy between PrEP eligibility and self-perceived HIV risk among men who have sex with men in China

|  | **Awareness (n=1085) ^a^** | | |  | **Acceptance (n=934) ^b^** | | |  | **Uptake (n=1085) ^a^** | | |
| --- | --- | --- | --- | --- | --- | --- | --- | --- | --- | --- | --- |
|  | **Total** | **Aware** |  |  | **Total** | **Accept** |  |  | **Total** | **Using/Ever used** |  |
|  | **n (%)** | **n (%)** | ***P^*^*** |  | **n (%)** | **n (%)** | ***P^*^*** |  | **n (%)** | **n (%)** | ***P^*^*** |
| **Age group (years)** |  |  | 0.512 |  |  |  | 0.098 |  |  |  | 0.396 |
| **18-25** | 177 (16.3) | 151 (85.3) |  |  | 152 (16.3) | 119 (78.3) |  |  | 177 (16.3) | 18 (10.2) |  |
| **26-35** | 535 (49.3) | 433 (80.9) |  |  | 459 (49.1) | 341 (74.3) |  |  | 535 (49.3) | 72 (13.5) |  |
| **36-45** | 254 (23.4) | 211 (83.1) |  |  | 222 (23.8) | 154 (69.4) |  |  | 254 (23.4) | 25 (9.8) |  |
| **>45** | 119 (11.0) | 95 (79.8) |  |  | 101 (10.8) | 67 (66.3) |  |  | 119 (11.0) | 16 (13.4) |  |
| **Residence** |  |  | <0.001 |  |  |  | <0.001 |  |  |  | 0.054 |
| **Urban** | 590 (54.4) | 526 (89.2) |  |  | 492 (52.7) | 403 (81.9) |  |  | 590 (54.4) | 84 (14.2) |  |
| **Town** | 271 (25.0) | 237 (87.5) |  |  | 242 (25.9) | 182 (75.2) |  |  | 271 (25.0) | 27 (10.0) |  |
| **Rural** | 224 (20.6) | 127 (56.7) |  |  | 200 (21.4) | 96 (48.0) |  |  | 224 (20.6) | 20 (8.9) |  |
| **Sexual orientation** |  |  | 0.940 |  |  |  | 0.157 |  |  |  | 0.225 |
| **Homosexual** | 771 (71.1) | 632 (82.0) |  |  | 658 (70.4) | 471 (71.6) |  |  | 771 (71.1) | 99 (12.8) |  |
| **Bisexual and other** | 314 (28.9) | 258 (82.2) |  |  | 276 (29.6) | 210 (76.1) |  |  | 314 (28.9) | 32 (10.2) |  |
| **Disclosure of sexual orientation to others** |  |  | <0.001 |  |  |  | <0.001 |  |  |  | 0.002 |
| **Yes** | 553 (51.0) | 503 (91.0) |  |  | 461 (49.4) | 396 (85.9) |  |  | 553 (51.0) | 83 (15.0) |  |
| **No** | 532 (49.0) | 387 (72.7) |  |  | 473 (50.6) | 285 (60.3) |  |  | 532 (49.0) | 48 (9.0) |  |
| **Marital status (with female)** |  |  | 0.929 |  |  |  | 0.602 |  |  |  | 0.083 |
| **Single** | 673 (62.0) | 554 (82.3) |  |  | 569 (60.9) | 417 (73.3) |  |  | 673 (62.0) | 91 (13.5) |  |
| **Married** | 327 (30.1) | 266 (81.3) |  |  | 288 (30.8) | 205 (71.2) |  |  | 327 (30.1) | 35 (10.7) |  |
| **Divorced/Widowed** | 85 (7.8) | 70 (82.4) |  |  | 77 (8.2) | 59 (76.6) |  |  | 85 (7.8) | 5 (5.9) |  |
| **Education** |  |  | <0.001 |  |  |  | <0.001 |  |  |  | 0.037 |
| **Middle school and below** | 149 (13.7) | 84 (56.4) |  |  | 131 (14.0) | 43 (32.8) |  |  | 149 (13.7) | 14 (9.4) |  |
| **High school/Junior college** | 277 (25.5) | 215 (77.6) |  |  | 252 (27.0) | 182 (72.2) |  |  | 277 (25.5) | 24 (8.7) |  |
| **University/College and above** | 659 (60.7) | 591 (89.7) |  |  | 551 (59.0) | 456 (82.8) |  |  | 659 (60.7) | 93 (14.1) |  |
| **Occupation** |  |  | <0.001 |  |  |  | <0.001 |  |  |  | 0.079 |
| **Freelancer** | 325 (30.0) | 226 (69.5) |  |  | 289 (30.9) | 173 (59.9) |  |  | 325 (30.0) | 30 (9.2) |  |
| **Corporate Employees** | 179 (16.5) | 151 (84.4) |  |  | 156 (16.7) | 110 (70.5) |  |  | 179 (16.5) | 21 (11.7) |  |
| **Individual Entrepreneurship** | 305 (28.1) | 278 (91.1) |  |  | 269 (28.8) | 233 (86.6) |  |  | 305 (28.1) | 35 (11.5) |  |
| **Government official** | 188 (17.3) | 158 (84.0) |  |  | 158 (16.9) | 115 (72.8) |  |  | 188 (17.3) | 28 (14.9) |  |
| **Unemployed/Retired/Student** | 88 (8.1) | 77 (87.5) |  |  | 62 (6.6) | 50 (80.6) |  |  | 88 (8.1) | 17 (19.3) |  |
| **Income (CNY/month)** |  |  | <0.001 |  |  |  | <0.001 |  |  |  | 0.515 |
| **<3000** | 196 (18.1) | 128 (65.3) |  |  | 166 (17.8) | 77 (46.4) |  |  | 196 (18.1) | 25 (12.8) |  |
| **3000-6000** | 481 (44.3) | 390 (81.1) |  |  | 421 (45.1) | 300 (71.3) |  |  | 481 (44.3) | 52 (10.8) |  |
| **>6000** | 408 (37.6) | 372 (91.2) |  |  | 347 (37.2) | 304 (87.6) |  |  | 408 (37.6) | 54 (13.2) |  |
| **HIV testing in the last 3 months** |  |  | 0.475 |  |  |  | 0.740 |  |  |  | 0.113 |
| **Yes** | 830 (76.5) | 677 (81.6) |  |  | 724 (77.5) | 526 (72.7) |  |  | 830 (76.5) | 93 (11.2) |  |
| **No** | 255 (23.5) | 213 (83.5) |  |  | 210 (22.5) | 155 (73.8) |  |  | 255 (23.5) | 38 (14.9) |  |
| **Other STI testing in the last 3 months** |  |  | 0.196 |  |  |  | 0.034 |  |  |  | 0.016 |
| **Yes** | 806 (74.3) | 654 (81.1) |  |  | 703 (75.3) | 525 (74.7) |  |  | 806 (74.3) | 86 (10.7) |  |
| **No** | 279 (25.7) | 236 (84.6) |  |  | 231 (24.7) | 156 (67.5) |  |  | 279 (25.7) | 45 (16.1) |  |
| **Diagnosed with STIs before** |  |  | <0.001 |  |  |  | 0.002 |  |  |  | <0.001 |
| **Yes** | 129 (11.9) | 120 (93.0) |  |  | 129 (13.8) | 64 (49.6) |  |  | 129 (11.9) | 39 (30.2) |  |
| **No** | 778 (71.7) | 612 (78.7) |  |  | 778 (83.3) | 484 (62.2) |  |  | 778 (71.7) | 75 (9.6) |  |
| **Not sure/Refuse to answer** | 178 (16.4) | 158 (88.8) |  |  | 178 (19.1) | 133 (74.7) |  |  | 178 (16.4) | 17 (9.6) |  |
| **Any current STI symptoms** |  |  | 0.108 |  |  |  | 0.562 |  |  |  | <0.001 |
| **Yes** | 77 (7.1) | 70 (90.9) |  |  | 44 (4.7) | 35 (79.5) |  |  | 77 (7.1) | 31 (40.3) |  |
| **No** | 998 (92.0) | 812 (81.4) |  |  | 881 (94.3) | 639 (72.5) |  |  | 998 (92.0) | 100 (10.0) |  |
| **Not sure/Refuse to answer** | 10 (0.9) | 8 (80.0) |  |  | 9 (1.0) | 7 (77.8) |  |  | 10 (0.9) | 0 (0) |  |
| **Ever used PEP for HIV prevention** |  |  | 0.002 |  |  |  | 0.310 |  |  |  | <0.001 |
| **Yes** | 147 (13.5) | 134 (91.2) |  |  | 44 (4.7) | 35 (79.5) |  |  | 147 (13.5) | 103 (70.1) |  |
| **No** | 938 (86.5) | 756 (80.6) |  |  | 890 (95.3) | 646 (72.6) |  |  | 938 (86.5) | 28 (3.0) |  |
| **Any sexual partners with HIV infection in the last 3 months** | | | 0.009 |  |  |  | 0.001 |  |  |  | <0.001 |
| **Yes** | 48 (4.4) | 46 (95.8) |  |  | 31 (3.3) | 24 (77.4) |  |  | 48 (4.4) | 16 (33.3) |  |
| **No** | 772 (71.2) | 619 (80.2) |  |  | 701 (75.1) | 489 (69.8) |  |  | 772 (71.2) | 60 (7.8) |  |
| **Not sure/Refuse to answer** | 265 (24.4) | 225 (84.9) |  |  | 202 (21.6) | 168 (83.2) |  |  | 265 (24.4) | 55 (20.8) |  |
| **Have sex with foreigners in the last 3 months** | | | 0.105 |  |  |  | 0.001 |  |  |  | <0.001 |
| **Yes** | 47 (4.3) | 44 (93.6) |  |  | 27 (2.9) | 22 (81.5) |  |  | 47 (4.3) | 18 (38.3) |  |
| **No** | 997 (91.9) | 813 (81.5) |  |  | 877 (93.9) | 646 (73.7) |  |  | 997 (91.9) | 107 (10.7) |  |
| **Not sure/Refuse to answer** | 41 (3.8) | 33 (80.5) |  |  | 30 (3.2) | 13 (43.3) |  |  | 41 (3.8) | 6 (14.6) |  |
| **Have sex with women in the last 3 months** | | | 0.380 |  |  |  | 0.038 |  |  |  | 0.003 |
| **Yes** | 132 (12.2) | 114 (86.4) |  |  | 100 (10.7) | 73 (73.0) |  |  | 132 (12.2) | 27 (20.5) |  |
| **No** | 919 (84.7) | 748 (81.4) |  |  | 810 (86.7) | 596 (73.6) |  |  | 919 (84.7) | 98 (10.7) |  |
| **Not sure/Refuse to answer** | 34 (3.1) | 28 (82.4) |  |  | 24 (2.6) | 12 (50.0) |  |  | 34 (3.1) | 6 (17.6) |  |
| **Number of male sexual partners in the past 3 months** | | | 0.008 |  |  |  | 0.012 |  |  |  | <0.001 |
| **<3** | 662 (61.0) | 524 (79.2) |  |  | 586 (62.7) | 412 (70.3) |  |  | 662 (61.0) | 59 (8.9) |  |
| **3-5** | 202 (18.6) | 176 (87.1) |  |  | 160 (17.1) | 116 (72.5) |  |  | 202 (18.6) | 40 (19.8) |  |
| **>5** | 221 (20.4) | 190 (86.0) |  |  | 188 (20.1) | 153 (81.4) |  |  | 221 (20.4) | 32 (14.5) |  |
| **Male sexual partner in the last sexual act** |  |  | <0.001 |  |  |  | <0.001 |  |  |  | 0.353 |
| **Regular sex partner (boyfriend)** | 374 (34.5) | 326 (87.2) |  |  | 317 (33.9) | 273 (86.1) |  |  | 374 (34.5) | 42 (11.2) |  |
| **Long-term fuck buddy** | 209 (19.3) | 192 (91.9) |  |  | 186 (19.9) | 175 (94.1) |  |  | 209 (19.3) | 21 (10.0) |  |
| **Casual or commercial sex partner** | 502 (46.3) | 372 (74.1) |  |  | 431 (46.1) | 233 (54.1) |  |  | 502 (46.3) | 68 (13.5) |  |
| **Usual frequency of alcohol drinking (per week)** | | | <0.001 |  |  |  | <0.001 |  |  |  | <0.001 |
| **Never** | 544 (50.1) | 404 (74.3) |  |  | 492 (52.7) | 320 (65.0) |  |  | 544 (50.1) | 48 (8.8) |  |
| **Sometimes (<3 times)** | 478 (44.1) | 429 (89.7) |  |  | 386 (41.3) | 320 (82.9) |  |  | 478 (44.1) | 80 (16.7) |  |
| **Always (≥3 times)** | 63 (5.8) | 57 (90.5) |  |  | 56 (6.0) | 41 (73.2) |  |  | 63 (5.8) | 3 (4.8) |  |
| **Ever used prohibited drugs** |  |  | 0.036 |  |  |  | 0.017 |  |  |  | <0.001 |
| **Yes** | 164 (15.1) | 144 (87.8) |  |  | 122 (13.1) | 102 (83.6) |  |  | 164 (15.1) | 40 (24.4) |  |
| **No** | 906 (83.5) | 731 (80.7) |  |  | 801 (85.8) | 571 (71.3) |  |  | 906 (83.5) | 89 (9.8) |  |
| **Not sure/Refuse to answer** | 15 (1.4) | 15 (100.0) |  |  | 11 (1.2) | 8 (72.7) |  |  | 15 (1.4) | 2 (13.3) |  |
| **Ever used prohibited drugs during last male-to-male sex** | | | 0.043 |  |  |  | 0.015 |  |  |  | 0.003 |
| **Yes** | 123 (11.3) | 109 (88.6) |  |  | 96 (10.3) | 80 (83.3) |  |  | 123 (11.3) | 25 (20.3) |  |
| **No** | 962 (88.7) | 781 (81.2) |  |  | 838 (89.7) | 601 (71.7) |  |  | 962 (88.7) | 106 (11.0) |  |
| **Self-perceived risk of HIV infection** |  |  | 0.015 |  |  |  | 0.370 |  |  |  | 0.105 |
| **High** | 269 (24.8) | 234 (87.0) |  |  | 226 (24.2) | 170 (75.2) |  |  | 269 (24.8) | 40 (14.9) |  |
| **Low** | 816 (75.2) | 656 (80.4) |  |  | 708 (75.8) | 511 (72.2) |  |  | 816 (75.2) | 91 (11.2) |  |

Abbreviations: CNY, Chinese Yuan; PEP, post-exposure prophylaxis; PrEP, pre-exposure prophylaxis; STI, sexually transmitted infections.

^a^for all those investigated; ^b^only for those have never used PrEP; ^*^Chi-square test or Fisher's exact method.

**Table S3.** Demographic and behavioural characteristics of MSM stratified by PrEP Eligibility and Self-Perceived HIV Risk (n=1085)

|  | **Risk discordant** | **Risk concordant** |  |
| --- | --- | --- | --- |
|  | **N (%)** | **N (%)** | ***P^*^*** |
| **Age group (years)** |  |  | 0.261 |
| **18-25** | 127 (71.8) | 50 (28.2) |  |
| **26-35** | 361 (67.5) | 174 (32.5) |  |
| **36-45** | 163 (64.2) | 91 (35.8) |  |
| **>45** | 74 (62.2) | 45 (37.8) |  |
| **Residence** |  |  | <0.001 |
| **Urban** | 376 (63.7) | 214 (36.3) |  |
| **Town** | 212 (78.2) | 59 (21.8) |  |
| **Rural** | 137 (61.2) | 87 (38.8) |  |
| **Sexual orientation** |  |  | <0.001 |
| **Homosexual** | 490 (63.6) | 281 (36.4) |  |
| **Bisexual and other** | 235 (74.8) | 79 (25.2) |  |
| **Disclosure of sexual orientation to others** |  |  | 0.950 |
| **Yes** | 370 (66.9) | 183 (33.1) |  |
| **No** | 355 (66.7) | 177 (33.3) |  |
| **Marital status (with female)** |  |  | 0.067 |
| **Single** | 458 (68.1) | 215 (31.9) |  |
| **Married** | 204 (62.4) | 123 (37.6) |  |
| **Divorced/Widowed** | 63 (74.1) | 22 (25.9) |  |
| **Education** |  |  | <0.001 |
| **Middle school and below** | 68 (45.6) | 81 (54.4) |  |
| **High school/Junior college** | 203 (73.3) | 74 (26.7) |  |
| **University/College and above** | 454 (68.9) | 205 (31.1) |  |
| **Occupation** |  |  | <0.001 |
| **Freelancer** | 199 (61.2) | 126 (38.8) |  |
| **Corporate Employees** | 109 (60.9) | 70 (39.1) |  |
| **Individual Entrepreneurship** | 238 (78.0) | 67 (22.0) |  |
| **Government official** | 109 (58.0) | 79 (42.0) |  |
| **Unemployed/Retired/Student** | 70 (79.5) | 18 (20.5) |  |
| **Income (CNY/month)** |  |  | 0.019 |
| **<3000** | 118 (60.2) | 78 (39.8) |  |
| **3000-6000** | 341 (70.9) | 140 (29.1) |  |
| **>6000** | 266 (65.2) | 142 (34.8) |  |
| **HIV testing in the last 3 months** |  |  | 0.005 |
| **Yes** | 573 (69.0) | 257 (31.0) |  |
| **No** | 152 (59.6) | 103 (40.4) |  |
| **Other STI testing in the last 3 months** |  |  | <0.001 |
| **Yes** | 568 (70.5) | 238 (29.5) |  |
| **No** | 157 (56.3) | 122 (43.7) |  |
| **Diagnosed with STIs before** |  |  | 0.357 |
| **Yes** | 79 (61.2) | 50 (38.8) |  |
| **No** | 526 (67.6) | 252 (32.4) |  |
| **Not sure/Refuse to answer** | 120 (67.4) | 58 (32.6) |  |
| **Any current STI symptoms** |  |  | 0.043 |
| **Yes** | 42 (54.5) | 35 (45.5) |  |
| **No** | 675 (67.6) | 323 (32.4) |  |
| **Not sure/Refuse to answer** | 8 (80.0) | 2 (20.0) |  |
| **Ever used PEP for HIV prevention** |  |  | 0.738 |
| **Yes** | 625 (66.6) | 313 (33.4) |  |
| **No** | 100 (68) | 47 (32.0) |  |
| **Any sexual partners with HIV infection in the last 3 months** | |  | 0.007 |
| **Yes** | 33 (68.8) | 15 (31.2) |  |
| **No** | 536 (69.4) | 236 (30.6) |  |
| **Not sure/Refuse to answer** | 156 (58.9) | 109 (41.1) |  |
| **Have sex with foreigners in the last 3 months** |  |  | 0.188 |
| **Yes** | 32 (68.1) | 15 (31.9) |  |
| **No** | 671 (67.3) | 326 (32.7) |  |
| **Not sure/Refuse to answer** | 22 (53.7) | 19 (46.3) |  |
| **Have sex with women in the last 3 months** |  |  | 0.102 |
| **Yes** | 79 (59.8) | 53 (40.2) |  |
| **No** | 626 (68.1) | 293 (31.9) |  |
| **Not sure/Refuse to answer** | 20 (58.8) | 14 (41.2) |  |
| **Number of male sexual partners in the past 3 months** | |  | 0.001 |
| **<3** | 434 (65.6) | 228 (34.4) |  |
| **3-5** | 121 (59.9) | 81 (40.1) |  |
| **>5** | 170 (76.9) | 51 (23.1) |  |
| **Male sexual partner in the last sexual act** |  |  | <0.001 |
| **Regular sex partner (boyfriend)** | 280 (74.9) | 94 (25.1) |  |
| **Long-term fuck buddy** | 162 (77.5) | 47 (22.5) |  |
| **Casual or commercial sex partner** | 283 (56.4) | 219 (43.6) |  |
| **Usual frequency of alcohol drinking (per week)** |  |  | 0.056 |
| **Never** | 382 (70.2) | 162 (29.8) |  |
| **Sometimes (<3 times)** | 304 (63.6) | 174 (36.4) |  |
| **Always (≥3 times)** | 39 (61.9) | 24 (38.1) |  |
| **Ever used prohibited drugs** |  |  | <0.001 |
| **Yes** | 83 (50.6) | 81 (49.4) |  |
| **No** | 631 (69.6) | 275 (30.4) |  |
| **Not sure/Refuse to answer** | 11 (73.3) | 4 (26.7) |  |
| **Ever used prohibited drugs during last male-to-male sex** | |  | <0.001 |
| **Yes** | 45 (36.6) | 78 (63.4) |  |
| **No** | 680 (70.7) | 282 (29.3) |  |

Abbreviations: CNY, Chinese Yuan; PEP, post-exposure prophylaxis; PrEP, pre-exposure prophylaxis; STI, sexually transmitted infections.

^*^Chi-square test or Fisher's exact method.
